# Aprimorando a Ressuscitação Cardiopulmonar

**DOI:** 10.36660/abc.20220900

**Published:** 2023-01-24

**Authors:** Paolo Blanco Villela

**Affiliations:** 1 Universidade Federal do Rio de Janeiro Rio de Janeiro RJ Brasil Universidade Federal do Rio de Janeiro, Rio de Janeiro, RJ – Brasil

**Keywords:** Parada Cardíaca, Reanimação Cardiopulmonar, Taxa de Sobrevida, Cardioversão Elétrica/métodos, Hospitalização, Treinamento com Simulação de Alta Fidelidade/métodos, Escala de Borg, Pesquisa em Educação de Enfermagem

A parada cardiorrespiratória (PCR) é o evento de maior gravidade na área da saúde e seu pronto reconhecimento com o consequente início das manobras de ressuscitação cardiopulmonar (RCP) são fundamentais para o retorno da circulação espontânea (RCE). Apesar da ausência de dados epidemiológicos robustos, uma metanálise com 141 estudos incluindo países da América do Norte, Europa, Ásia e Oceania, demonstrou taxa de RCE abaixo de 30% nos casos de PCR extra-hospitalar, e concluiu que as chances de sucesso estavam ligadas ao fato da PCR ter sido testemunhada e em casos cuja RCP havia sido iniciada por um espectador da mesma.^1^

No Brasil, de acordo com a diretriz sobre Ressuscitação Cardiopulmonar e Cuidados Cardiovasculares de Emergência da Sociedade Brasileira de Cardiologia, as taxas de sobrevida de PCR extra-hospitalar, quando abordada dentro dos primeiros 5 minutos com desfibrilação precoce, variam entre 50% e 70%.^2^ Por outro lado, no ambiente intra-hospitalar, paradoxalmente, as taxas de sobrevida são inferiores a 20%, provavelmente devido às condições clínicas associadas e à maior gravidade dos pacientes, que fazem da assistolia e da atividade elétrica sem pulso os ritmos mais prevalentes.^2^

Independentemente do ambiente, os protocolos nacionais^2^ e internacionais^3^ para a abordagem da PCR ressaltam a importância da execução da cadeia de sobrevivência, cuja sequência de ações envolve a RCP de alta qualidade. Por sua vez, promover uma RCP de alta qualidade envolve minimizar interrupções nas compressões torácicas, promover compressões torácicas com frequência e profundidade adequadas, permitir retorno total do tórax entre as compressões e evitar a hiperventilação.^4^

Desta forma, as taxas de sucesso da RCP estão ligadas diretamente à correta execução das manobras e ao cumprimento dos protocolos de atendimento à PCR,^2-4^ o que requer treinamento e aprendizado constantes. Neste cenário, os mecanismos de *feedback* são cruciais para o aprimoramento das ações e devem fazer parte do conjunto de medidas que visam aumentar a sobrevida após uma PCR. Estes mecanismos são compostos por ações ou dispositivos que avaliam a qualidade da RCP e podem ser aparelhos eletrônicos, questionários, monitoramento por vídeo, supervisão humana ou qualquer outra estratégia com o mesmo objetivo. Abella et al.,^5^demostraram, por exemplo, em estudo observacional com 67 pacientes, que as manobras de RCP nos primeiros cinco minutos não atingiram as metas das diretrizes internacionais, com compressões lentas (<90bpm) e superficiais (<38mm). Estes achados foram possíveis devido ao uso de um dispositivo de *feedback* durante o atendimento a uma vítima de PCR intra-hospitalar.

Em outro estudo, Cheng et al.,^6^ utilizaram monitoramento em tempo real das manobras de RCP intra-hospitalar e as ações eram revistas e apresentadas em *feedback* semanal aos membros dos times de ressuscitação.^6^ Zheng et al.,^7^ por meio de pesquisa com 1405 profissionais de 75 hospitais terciários na China, observaram que 91% reconheciam a necessidade de monitoramento da RCP e 67,3% entendiam que o principal problema durante a RCP era a qualidade das compressões torácicas, influenciada diretamente pela fadiga do operador.^7^ Esta influência da fadiga foi também observada por Trowbridge et al.,^8^ que demonstraram redução significativa da profundidade das compressões torácicas dentro dos dois primeiros minutos do início das manobras de RCP, ressaltando a importância do revezamento de socorristas.^8^

Neste contexto, o artigo publicado na revista Arquivos Brasileiros de Cardiologia, traz informações importantes que podem contribuir para o aperfeiçoamento das equipes de atendimento à PCR.^9^ Nele, os autores combinaram a utilização de um dispositivo de *feedback* com a escala de Borg^10^para a percepção do cansaço durante a realização de manobras de RCP, em hospital público de ensino da cidade de São Paulo (SP – Brasil).^9^ O estudo foi realizado utilizando cenário simulado com auxílio de manequins com dispositivo de *feedback*, incluiu 69 enfermeiros de áreas críticas e não críticas de atendimento à adultos do hospital e todos os participantes utilizaram frequencímetro para registro da frequência cardíaca durante a atividade.^9^

Os autores observaram que os enfermeiros que efetuaram a RCP com dispositivo de *feedback* apresentaram menores médias na escala de Borg no primeiro e no segundo minutos quando comparados ao grupo controle (13,5 e 14,2 *versus* 14 e 14,9 respectivamente).^9^ Em relação à frequência cardíaca, observou-se comportamento semelhante, isto é, aqueles que realizaram a RCP em manequins com dispositivo de *feedback* apresentaram menores médias no primeiro e no segundo minutos em relação ao grupo controle (119 bpm e 121 bpm *versus* 121 bpm e 123 bpm).^9^ Apesar dos grupos apresentarem valores próximos, é importante destacar a tendência de apresentação, e quando combinados com a escala de Borg,^10^ permitem a mensuração de um dado objetivo, como a frequência cardíaca, em concomitância com um dado subjetivo, como a percepção de cansaço.

Apesar do número reduzido de participantes, do menor percentual de praticantes de atividade física no grupo controle (33,3% *versus *41,6%), e do fato da RCP ter sido realizada em manequim,^9^ os resultados do estudo demonstram com clareza a percepção de fadiga do operador, que é provavelmente o principal determinante do sucesso durante a realização da RCP. Além deste fato, Gandolfi et al.,^11^ em estudo semelhante, também em ambiente simulado e utilizando manequins com dispositivo de *feedback*, observaram melhora imediata da frequência e da profundidade das compressões torácicas quando o dispositivo era utilizado,^11^ permitindo aprimoramento em tempo real das manobras de RCP. Portanto, pode-se observar que o reconhecimento das limitações na execução das ações é fundamental para a realização das correções necessárias, e assim, os dispositivos de *feedback* podem impactar positivamente na qualidade da RCP.

Assim, torna-se cada vez mais relevante a discussão sobre o uso de mecanismos de *feedback* durante a assistência a uma vítima de PCR, e o presente estudo^9^ tem o mérito de centralizar a atenção no socorrista, demonstrando a importância do dispositivo na sua percepção de cansaço combinada à sua resposta ao exercício, ambas menores em relação ao grupo controle. Por fim, é importante ressaltar a necessidade de divulgação dos resultados de estudos neste cenário para que os dados sejam transformados em ações e a história natural da PCR seja modificada de forma consistente.
