# Supplementary material for: Aprimorando a Ressuscitação Cardiopulmonar
Source: Arq Bras Cardiol. 2023 Jan 24;120(1):e20220900. [Article in Portuguese] doi: 10.36660/abc.20220900 (PMC10389115; doi:10.36660/abc.20220900)
Supplement: Translation

# Translation: Improving Cardiopulmonary Resuscitation

**Authors:** Paolo Blanco Villela

**Affiliations:** 1 Brazil Universidade Federal do Rio de Janeiro, Rio de Janeiro, RJ – Brazil

**Keywords:** Heart Arrest, Cardiopulmonary Ressuscitation, Survival Rate, Eletric Countershock/methods, Hospitalization, Borg Scale, High Fidelity Simulation Training/methods, Nursing Education Research

Cardiopulmonary arrest (CRA) is the most serious event in the health area and its prompt recognition with the consequent initiation of cardiopulmonary resuscitation (CPR) maneuvers are essential for the return of spontaneous circulation (ROSC). Despite the absence of robust epidemiological data, a meta-analysis of 141 studies including countries in North America, Europe, Asia and Oceania, demonstrated a ROSC rate below 30% in cases of out-of-hospital CA, and concluded that the chances of success were linked to the fact that the CPR was witnessed and in cases where CPR had been initiated by a bystander.^1^

In Brazil, according to the guideline on Cardiopulmonary Resuscitation and Emergency Cardiovascular Care of the Brazilian Society of Cardiology, the survival rates of out-of-hospital CPA, when addressed within the first 5 minutes with early defibrillation, vary between 50% and 70% two.^2^ On the other hand, in the in-hospital environment, paradoxically, survival rates are less than 20%, probably due to associated clinical conditions and the greater severity of patients, which make asystole and pulseless electrical activity the most prevalent rhythms.^2^

Regardless of the environment, national^2^ and international^3^ protocols for approaching CRA emphasize the importance of executing the chain of survival, whose sequence of actions involves high-quality CPR. In turn, promoting high-quality CPR involves minimizing interruptions in chest compressions, providing chest compressions with adequate frequency and depth, allowing full chest recoil between compressions, and avoiding hyperventilation.^4^

In this way, the success rates of CPR are directly linked to the correct execution of the maneuvers and compliance with the CRA care protocols,^2-4^ which requires constant training and learning. In this scenario, feedback mechanisms are crucial for improving actions and should be part of the set of measures aimed at increasing survival after cardiac arrest. These mechanisms are composed of actions or devices that assess the quality of CPR and can be electronic devices, questionnaires, video monitoring, human supervision or any other strategy with the same objective. Abella et al.^5^ demonstrated, for example, in an observational study with 67 patients, that CPR maneuvers in the first five minutes did not reach the goals of international guidelines, with slow compressions (<90bpm) and superficial compressions (<38mm).

In another study, Cheng et al.^6^ used real-time monitoring of in-hospital CPR maneuvers and the actions were reviewed and presented in weekly feedback to members of the resuscitation teams.^6^ Zheng et al.,^7^ through a survey of 1405 professionals from 75 tertiary hospitals in China, observed that 91% recognized the need for CPR monitoring and 67.3% understood that the main problem during CPR was the quality of chest compressions, directly influenced by operator fatigue.^7^ This influence of fatigue was also observed by Trowbridge et al.,^8^ who demonstrated a significant reduction in the depth of chest compressions within the first two minutes of starting CPR maneuvers, highlighting the importance of alternating rescuers.^8^

In this context, the article published in the *Arquivos Brasileiros de Cardiologia* journal, brings important information that can contribute to the improvement of CRA care teams.^9^ In it, the authors combined the use of a feedback device with the Borg scale^10^ for the perception of tiredness while performing CPR maneuvers in a public teaching hospital in the city of São Paulo (SP – Brazil).^9^ The study was carried out using a simulated scenario with the aid of mannequins with a feedback device, including 69 nurses from critical and non-critical areas of adult care at the hospital and all participants used a frequency meter to record heart rate during the activity.^9^

The authors observed that nurses who performed CPR with a feedback device had lower means on the Borg scale in the first and second minutes when compared to the control group (13.5 and 14.2 versus 14 and 14.9 respectively).9 Regarding heart rate, a similar behavior was observed, that is, those who performed CPR on mannequins with a feedback device had lower averages in the first and second minutes compared to the control group (119 bpm and 121 bpm versus 121 bpm and 123 bpm).^9^ Although the groups present similar values, it is important to highlight the presentation tendency, and when combined with the Borg scale,^10^ they allow the measurement of objective data, such as heart rate, concomitantly with subjective data, such as the perception of tiredness.

Despite the reduced number of participants, the lower percentage of physical activity practitioners in the control group (33.3% versus 41.6%), and the fact that CPR was performed on a dummy,^9^ the results of the study clearly demonstrate the perception operator fatigue, which is probably the main determinant of success when performing CPR. In addition to this fact, Gandolfi et al. in a similar study, also in a simulated environment and using mannequins with a feedback device, they observed an immediate improvement in the frequency and depth of chest compressions when the device was used,^11^ allowing real-time improvement of CPR maneuvers. Therefore, it can be observed that the recognition of limitations in the execution of actions is fundamental for carrying out the necessary corrections, and thus, feedback devices can positively impact the quality of CPR.

Thus, the discussion on the use of feedback mechanisms when assisting a CRA victim becomes increasingly relevant, and the present study^9^ has the merit of focusing attention on the rescuer, demonstrating the importance of the device in their perception of fatigue combined with their response to exercise, both lower than the control group. Finally, it is important to emphasize the need to disseminate the results of studies in this scenario so that the data are transformed into actions and the natural history of CRP is consistently modified.
